# A tripartite game analysis of industrial structure upgrading and green development of regional economy: A case study of Shanxi Province, China

**DOI:** 10.1016/j.heliyon.2023.e20729

**Published:** 2023-10-13

**Authors:** Fuqiang Hu, Kwisik Min, Chengmeng Li, Bodong Song

**Affiliations:** a Southwest Forestry University, Kunming, 650224, China; b Hanyang University, Seoul, 02581, South Korea; c Gachon University, Seongnam, 13120, South Korea

**Keywords:** Evolutionary game, Industrial structure upgrading, Regional economy, Green development

## Abstract

In the contemporary context, both the upgrading of the industrial structure and the implementation of environmentally sustainable practices within the regional economy have emerged as central avenues for achieving quality development. This study examines the strategic behavior of local governments, capital, and people through the construction of a tripartite evolutionary game model. Subsequently, six different evolutionary stable strategy (ESS) are subjected to a comprehensive analysis. Finally, the parameters influencing the strategic decisions of each party are meticulously examined through simulation. The results of this study can be summarized as follows: First, it is shown that under appropriate conditions, all three entities support the scenario of stable development prospects associated with industrial structure upgrading (1, 1, 1). Second, the strategic choices made by capital and people depend on several factors, including existing profits, future benefits, and the costs associated with transformation. At the same time, local governments show a propensity to adopt incentive strategies. Ultimately, the research underscores the pronounced impact of future benefits, transformation costs, and the probability of success in industrial upgrading on all stakeholders, shaping their evolutionary trajectories and results. In particular, the probability of successful industrial structure upgrading exerts the greatest influence on evolutionary trajectories, while the possibility of government imposing carbon taxes and initial willingness primarily determine the evolutionary trajectory. This paper attempts to provide a new perspective on industrial structure upgrading and green development of the regional economy by combining evolutionary game theory and scenario analysis methods to promote the process of industrial structure upgrading and sustainable development.

## 1 Introduction

China's economic landscape has entered a new phase marked by a transition from rapid growth to higher-quality growth. A primary goal of the government is to facilitate the advancement of industrial structure, aiming at sustainable development within regional economies. Upgrading the industrial structure is the key to China's transformation and development [1]. Comprehensively implementing a strategy of regional coordinated development, consolidating the foundation of regional development, rationalizing regional economic relations, and promoting sustainable regional economic development are basic requirements and integral parts of quality development. Upgrading the industrial structure involves increasing the proportion of low-carbon or zero-emission ecological productivity in the regional economy and transforming resource-based industries into new sectors, such as the cultural tourism industry [2].

The upgrading of the industrial structure involves the progression from a lower to a higher configuration. Specifically, it involves a continuous shift of the national economic focus toward the tertiary sector, along with the expansion of the scale of tertiary industry, leading to the transformation of a low-value-added, labor-intensive industrial chain into a high-value-added counterpart [3]. Developing the industrial structure is a prerequisite for achieving high-quality economic growth [4]. Therefore, at this stage, the transition from a resource-dependent economy to a cultural and tourism-oriented industry emerges as a key avenue for effectively facilitating the advancement of industrial structure [5]. Reflecting on the strategies for realizing this course of action plays a key role in realizing the high-quality growth of China's regional economy. In addition to upgrading the industrial structure, sustainable development is imperative in the current context of socio-economic progress. The Chinese government has adopted a “carbon peaking and carbon neutrality” strategy, which requires the country's economy to make an environmentally friendly transition [6]. Shifting from a resource-dependent economy to a cultural tourism-oriented industry can not only facilitate the advancement of the industrial structure of the regional economy but also lead to the ecologically sound transformation of the regional economy [7,8]. As a result, the realization of high-quality development in China's regional economy, along with the improvement of industrial structure and the pursuit of sustainable development, takes on profound significance [9].

While upgrading the industrial structure is of paramount importance for the current stage of China's economic development, it is discouraging that under the system of “Chinese-style decentralization” and “promotion tournament” for officials, local governments lack the motivation to effectively implement environmental management and promote local industrial upgrading [10]. Driven by self-interest, local governments may prioritize short-term gains at the expense of environmental and industrial upgrading opportunities. While relying on resources to drive economic development is relatively easy due to mature resource extraction and processing technologies and a well-developed industrial chain, the transition to cultural tourism requires substantial investment with minimal short-term economic returns [11]. From a capital perspective, investment in the cultural tourism industry requires significant capital, lacks experience, carries inherent risks, and does not guarantee stable returns comparable to those from investments in resource-based industries. In addition, local people face the challenge of changing long-established habits that span decades or even centuries. People's production methods, work styles, and even aspects of daily life such as medical care and education are closely tied to resource-based industries. As a result, there is a degree of resistance to the transformation and upgrading of the industrial structure.

Shanxi Province in China serves as a typical example [12]. The province exhibits a high proportion of secondary industry, a limited and sluggish share of tertiary industry, and a significant lack of coordination in the development of these three sectors, which manifests in the province's industrial structure. With its heavy reliance on the coal industry, Shanxi Province lacks alternative industries to ensure stable economic development. As a major coal-producing region, Shanxi is projected to produce 1.19 billion tons of raw coal in 2021, supplying 43.56 million tons of coal to 16 provinces and cities [13]. This coal-dependent growth trend is evident. The secondary industry, particularly coal and other heavy industries, remains the primary driving force behind Shanxi's economic development [14]. However, in recent years, several factors have contributed to the decline in the growth rate of non-coal GDP in Shanxi Province., while other major provinces across the country have experienced steady and continuous socioeconomic development [15]. Consequently, Shanxi Province finds itself in an uncomfortable position regarding its development. Furthermore, the long-term mining, transportation, and utilization of coal resources have given rise to deep-rooted conflicts. This type of development, characterized by rapid growth in a single economy followed by a decline due to its limitations, now poses the greatest obstacle to Shanxi Province's economic progress [16]. On the other hand, Shanxi Province boasts abundant cultural and tourism resources, encompassing numerous historical and cultural sites [17]. Its geographical location in the transition zone between the second and third steps of the Chinese economic ladder, characterized by a topography of “two mountains sandwiched by a river,” has resulted in the creation of many scenic spots [18,19]. The province possesses a wealth of both natural and humanistic landscapes, indicating promising prospects for the development of the cultural tourism industry [17]. Many regions in China face similar circumstances to Shanxi, thereby making the upgrading of the industrial structure of resource-based economies and achieving high-quality regional economic development a focal point of attention.

Based on the above background, in this context, it is crucial to explore the evolutionary stable strategy (ESS) adopted by various stakeholders, including local governments, capital, and the general public, following a comprehensive evolutionary game. This research aims to identify the key factors influencing ESSs, thereby facilitating the achievement of industrial structure upgrading in resource-based regional economies, enabling local governments, investors, and the public to maximize their respective interests, and promoting high-quality regional economic development and sustainable regional growth. In line with our research objectives, this study seeks to address the following three questions: (i) What methods can accurately assess the benefits and drawbacks experienced by local governments, capitals, and the general people within the framework of the tripartite evolutionary game model? How can we appropriately formulate the payment matrix to reflect these dynamics? (ii) How can we analyze the asymptotic stability of all three parties involved? What equilibrium strategies can we observe among them, and under what circumstances can these strategies be realized? (iii) To what extent do certain crucial parameters influence the outcomes and evolutionary trajectories in this context?

To address these questions, this paper initially presents a set of rational assumptions to effectively characterize the benefits and costs associated with local governments, capitalists, and the general public, considering the actual circumstances. Then, the payment matrix is constructed and dynamic equations are formulated within the framework of the evolutionary game model. A comprehensive analysis is then performed to investigate the asymptotic stability of the three parties and the asymptotic stability of the evolutionary stable strategies (ESSs) under various stability conditions. Finally, numerical simulations are performed to investigate the ESSs and to identify the key parameters that impact the results, such as the costs associated with changing the lifestyles and production methods of individuals and capitalists, government subsidies, future benefits to the people, immediate benefits to capitals in the resource-based and cultural tourism industries, and initial willingness. These simulations provide theoretical insights and serve as a valuable reference for all stakeholders seeking optimal strategies.

This paper makes notable contributions to the existing literature in two key areas. First, it addresses the pressing issues of industrial upgrading and sustainable development in regional economies in the Chinese context. In particular, it analyzes the dynamic interplay between local governments, capitalists, and the public. Second, this research extends the use of the evolutionary game model. To our current understanding, this is the first instance in which the evolutionary game model is used to examine the tripartite decision-making process related to industrial structure upgrading in Chinese provinces heavily reliant on resource-based industries. By investigating the asymptotic stability demonstrated by the three parties and identifying Equilibrium Strategy Selections (ESSs) under different stability conditions, along with analyzing the impact of crucial parameters on outcomes and evolutionary paths, this paper provides invaluable theoretical guidance to all stakeholders. The aim is to facilitate the transition to industrial upgrading and promote sustainable development in regional economies. Ultimately, the goal is to achieve a stable strategy in which all parties support industrial upgrading and maximize their respective benefits throughout the process.

## 2 Literature review

In light of the aforementioned objectives, this paper conducts a review of the existing literature on industrial structure upgrading for regional economic green development and evolutionary game models.

### 2.1 Industrial structure upgrading

Most current studies on industrial structure upgrading and green development of regional economies are based on empirical research methods. Regarding the significance of industrial structure upgrading, scholars widely agree that it is crucial to approach it scientifically and accurately in line with economic development [[20], [21], [22]]. Researchers have approached this issue from different perspectives. There is a correlation between technological progress bias, industrial restructuring, and the dynamics of regional industrial economic growth [20]. Technological progress plays a role in promoting industrial restructuring, and different technologies have different effects on industrial restructuring and upgrading [23]. Industrial structure upgrading involves reducing carbon emissions without sacrificing economic development [24]. Positive effects on industrial structure upgrading are observed in the pilot projects of low-carbon cities [21]. Financial development and China's foreign direct investment contribute to promoting industrial structure upgrading [25]. The development of green finance is closely related to the strengthening of the tertiary sector, which plays an important role in industrial structure upgrading [26]. In addition, the development of the digital economy facilitates industrial structure upgrading and helps achieve green development in regional economies [27].

Some scholars mainly study the influence of policies, regulations, and government management on industrial structure upgrading and green development in regional economies. Governments implement relevant policies to promote industrial structure upgrading, with economic measures being particularly effective [22]. The level of government management varies among Chinese provinces, resulting in different approaches to achieving industrial structure upgrading. As a result, government management cannot be generalized [28,29]. Environmental regulations set by governments can promote technological reform and industrial upgrading to facilitate the green development of regional economies [30], but the impact of industrial upgrading depends greatly on the management goals pursued [31]. Local government policies, especially those aligned with business interests, can better support regions in upgrading their industrial structures, although the effects may vary from region to region [1]. The interaction of local government management also influences industrial structure upgrading to varying degrees, with a positive effect observed mainly in economically developed regions due to regional disparities [32]. Financial disparities between different local governments may hinder the smooth process of industrial structure upgrading [33].

### 2.2 Evolutionary game theory

Evolutionary game theory came to prominence in the 1990s, merging classical game theory with Darwin's biological theory of evolution. Unlike traditional game theory, evolutionary game theory doesn't assume perfect rationality or complete information. Rather, it views entities as participants with limited rationality who engage in processes of learning, strategic adaptation, and repeated interactions to optimize their respective interests [34]. In addition, evolutionary game theory places a strong emphasis on dynamic equilibrium and is a valuable framework for examining interactions within complex systems while tracing the evolutionary paths of individual strategies [35,36].

This theory has been widely applied to decision-making processes within political parties. Scholars have conducted game analyses focusing on the relationship between central and local governments. They have observed that local governments are often less motivated in these games, particularly in the context of environmental monitoring systems [37]. In the area of smart manufacturing, researchers have developed evolutionary game models to study the interactions between the government and manufacturing firms. They have studied the effects of government subsidies and penalties on incentives for firms to adopt smart manufacturing practices [38]. To study the interplay between enterprise investment and employee behavior, researchers have constructed a two-sided evolutionary game model that takes into account the security investments made by enterprises and the corresponding behaviors of employees. This model enhances the accuracy of corporate decision-making by evaluating the costs and benefits associated with safety investments [39]. Similarly, in the field of developing high-quality manufacturing industries, scholars have formulated a three-party evolutionary game model involving the local government, leading enterprises, and follower enterprises. They have analyzed the influence of various factors, such as initial probability and innovation capability, on the results of this game [40]. Similarly, another study established a three-party evolutionary game model involving local governments, power companies, and coal companies to explore the evolutionary path of cleaner production in the context of coal overcapacity. In the area of supervision, one study introduced the role of whistleblowers and constructed a three-way evolutionary game model involving regulators, firms, and whistleblowers. This model simulated two ideal evolutionary stability points and revealed a significant correlation between whistleblowing, management, and supervision [41].

### 2.3 Literature gaps

After a comprehensive review of the existing literature, we identified four major gaps. First, in the area of industrial upgrading, most studies predominantly use empirical methods to analyze the impact of specific local government policies. However, the intricate interplay of policies involving stakeholders has received very little attention. Moreover, the use of evolutionary game methods for modeling, simulation, and analysis remains relatively unexplored territory. Second, current scholarship on industrial upgrading focuses primarily on comparative analyses across regions, with little attention paid to the specifics of individual provinces heavily dependent on resource-based industries. Third, despite the widespread use of evolutionary game theory in the context of multi-party decision-making for supervisory purposes, the incorporation of this model into the domain of industrial structure upgrading remains conspicuously absent from the literature. Fourth, a significant proportion of the existing studies are limited to the examination of the interaction between the levels of government and business enterprises, with limited attention paid to the public perspective. Paradoxically, the role of the public in shaping industrial upgrading is crucial and warrants greater study. Moreover, the study of the success rate of industrial upgrading as an influential benchmark remains underrepresented in scholarly discourse.

To address these gaps, this paper makes two important contributions. First, it provides an insightful analysis of the reality of industrial restructuring in Shanxi Province, a prominent resource-based industrial region in China. It thoroughly examines the roles played by the local government, capitalists, and the general public in this process. Second, this paper pioneers the application of the evolutionary game model to investigate the challenges of industrial structure upgrading and regional economic green development in a specific Chinese province. The analysis delves deeply into the asymptotic stability of each participant and the stability of the Evolutionary Stable Strategies (ESS) under various conditions. This is achieved through the formulation of hypotheses, the construction of payoff matrix, and the establishment of replicated dynamic equations. Furthermore, numerical simulations are conducted to explore the six ESS scenarios and identify the key parameters that affect the evolutionary results and trajectories. By providing policy insights, this study aims to promote the support of industrial structure upgrading and green development among all stakeholders, thus maximizing the benefits for all parties involved.

## 3 Methodology

### 3.1 Basic assumptions

The main drivers of regional industrial upgrading and green development are the support and decisions of the main actors of the social economy: the capitalists. However, it is important to recognize that the government also plays an important role in this process as an environmental creator. By implementing an “incentive” strategy, the government provides policy guidance and financial support to facilitate the transition from resource-based industries to the development of the cultural tourism sector, thus promoting the stable growth of the regional economy. In addition, the active cooperation of the public is crucial to the realization of industrial upgrading, as it requires a change in work and lifestyle. Alternatively, if the government adopts a " non-incentive " strategy, the development of capitalists and individuals takes place within the framework of market forces. In the context of the evolutionary game, participants exhibit limited rationality and undergo dynamic evolution, reflecting the decision-making behavior of individuals and the influence of the external environment.

**Fig. 1:**
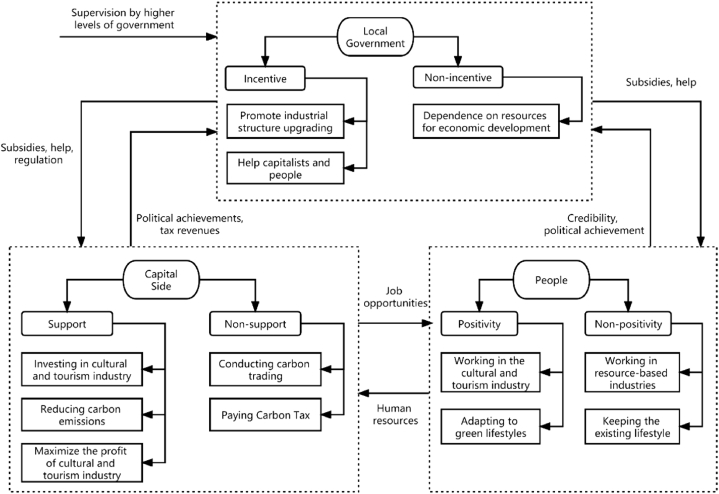
The relationship and strategy set between the players.

Fig. 1 illustrates the interaction between the local government, capital, and the people, as discussed in previous analyses. Given the multi-stage and dynamic nature of regional industrial structure upgrading, this paper adopts a hybrid mechanism perspective to study the dynamic evolutionary game involved. Based on this approach, the following hypotheses are formulated to guide the research.

Assumption 1 The evolutionary game of industrial structure upgrading involves three key parties: the local government, capital, and the people. It is postulated that in this study, local government, capital, and people at all levels are assumed to be uniform, which means there is no significant heterogeneity among them. In terms of upgrading the industrial structure and high-quality development of the regional economy, local governments serve as the main leaders and managers of this development. At the same time, the profitability of capital investments follows a relatively long cycle, which allows them to make continuous adjustments in response to prevailing circumstances. At the same time, changes in the lifestyles and work patterns of the population involve a longer-term process. Individuals tend to make strategic adjustments in response to the economic landscape, employment status, and prospects of industries. All parties involved adhere to the assumption of finite rationality, learning from each other, imitating each other, and evolving within the game process to maximize their interests [42].

Assumption 2 The local government's strategy set consists of {incentive, non-incentive}. The incentive strategy involves the adoption by the local government of policies and the allocation of resources aimed at encouraging the active participation of both capitalists and the general population in the effort to improve the industrial structure. The probability that the local government chooses the incentive strategy is denoted as x (x∈[0,1]), while the probability that it chooses the discourage strategy is 1−x. Similarly, the capitalist's strategy set includes {support, non-support}. The support strategy indicates that the capital will invest and operate in the cultural tourism industry with a probability denoted as y (y∈[0,1]). Conversely, the capital's probability of choosing the non-support strategy is 1−y. The strategy set for the people is set to {positivity, non-positivity}, where the positive strategy means the active adoption of lifestyle and work style changes to facilitate the upgrading of the regional industrial structure and promote green development. The probability of choosing the positivity strategy is denoted by (z∈[0,1]), while the probability of choosing the non-positivity strategy is 1−z. Given the complex nature of industrial upgrading and regional economic green development, the potential for failure is an important consideration. Therefore, this paper introduces the variable " a" to represent the probability of successful industrial structure upgrading.

Assumption 3 If the local government chooses the incentive strategy, it will receive future benefits from green development, denoted by $F_{1}$. If the industrial structure upgrading is successful, the government can receive credibility, tax, and performance benefits from both the capital and the people, denoted by $R_{12}$ and $R_{13}$, respectively. In this scenario, should the capitalist choose the support strategy, the government will enable the capitalist to proceed accordingly. Conversely, if the capitals choose the non-support strategy, the government will impose a carbon tax with a probability represented by b, denoted by S. Similarly, if the people choose the positivity strategy, the government will provide them with subsidies, denoted as $C_{13}$. On the other hand, if the government adopts the non-incentive strategy and allows an increased reliance on resource-based industries in the regional economy, it will suffer a loss of credibility and accountability from higher-level entities, denoted by $P_{1}$.

Assumption 4 If the capital chooses the non-support strategy, the current income is equal to the income generated by the resource-based industry, denoted as $R_{21}$. In this case, the industry must emit a greater amount of carbon and is obligated to pay a carbon trading fee, represented by $M_{1}$. Conversely, if the capital chooses the support strategy, the current income comes from the cultural tourism industry, denoted as $R_{22}$. At this point, the capital is entitled to future income from green development, denoted as $F_{2}$, but due to the shorter development time of the culture and tourism industry, it requires an up-front investment, denoted as $C_{21}$.

Assumption 5 If people choose the non-positivity strategy, they will be employed by resource-based companies and earn $R_{31}$. Conversely, if people choose the positivity strategy, they will work in the culture and tourism industry and earn $R_{32}$. Given the increasing importance of the culture and tourism industry in the future, the people will receive the future benefit $F_{3}$. However, there is a cost associated with the transition from their old way of living and working, denoted as $C_{3}$.

Each of the notations and their respective meanings are shown in Table 1.

**Table 1:** Description of parameters in the evolutionary game model.

Parameters	Description
a	Probability of success in upgrading industrial structures
$F_{1}$	Future benefits of industrial structure upgrading for local government
$R_{12}$	Government benefits from capital when choosing incentive strategies
$C_{12}$	Government subsidies for capital
b	Probability of the government collecting a carbon tax
S	The amount of carbon tax collected by the government
$R_{13}$	Government benefits from the people when choosing incentive strategies
$C_{13}$	Government subsidies for people
$P_{1}$	Government receives punishment from higher levels of government and loss of credibility when it chooses non-incentive strategy
$R_{22}$	The current income of the capital to operate the cultural tourism industry
$F_{2}$	Future income of capital after industrial structure upgrade
$C_{21}$	Transformation costs paid by capital when choosing support strategy
$R_{21}$	Current income of resource-based industries operated by the capital
$M_{1}$	Carbon trading fees when capital choose non-support strategy
$R_{32}$	Current income of the people in the cultural tourism industry
$F_{3}$	Future income of people after industrial structure upgrade
$C_{3}$	The cost to the people for changing the way they live and work
$R_{31}$	Current people's income in resource-based industries
x	Probability of local government choosing the incentive strategy
1−x	Probability of local government choosing the non-incentive strategy
y	Probability of capital choosing the support strategy
1−y	Probability of capital choosing the non-support strategy
z	Probability of people choosing the positivity strategy
1−z	Probability of people choosing the non-positivity strategy

### 3.2 Evolutionary game model

The payoff matrix of local government, capital and people is shown in Table 2.

**Table 2:** Payoff matrix of local government, capital and people.

Stakeholders				People	
				Positivity (z)	Non-positivity (1−z)
Local government	Incentive (x)	Capital side	Support (y)	$aF_{1}+R_{12}+R_{13}-C_{12}-C_{13}$,	$R_{12}-C_{12}$,
				$R_{22}+{aF}_{2}+C_{12}-C_{21}$*,*	$R_{22}+aF_{2}+C_{12}-C_{21}$,
				$C_{13}+R_{32}+aF_{3}-C_{3}$	$R_{31}$
			Non-support (1−y)	$bS+R_{13}-C_{13}$,	$bS+aF_{1}$,
				$R_{21}-M_{1}-bS$,	$R_{21}-M_{1}-bS$,
				$C_{13}+R_{32}+aF_{3}$ $-C_{3}$	$R_{31}$
	Non-incentive (1−x)	Capital side	Support (y)	$-P_{1}$,	$-P_{1}$,
				$R_{22}+aF_{2}-C_{21}$,	$R_{22}+aF_{2}-C_{21}$,
				$R_{32}+aF_{3}-C_{3}$	$R_{31}$
			Non-support (1−y)	$-P_{1}$,	$-P_{1}$,
				$R_{21}-M_{1}$,	$R_{21}-M_{1}$,
				$R_{32}+aF_{3}-C_{3}$	$R_{31}$

Assuming that the expected utility of the incentive strategy of the local government is $U_{11}$, the expected utility of the non-incentive strategy of the local government is $U_{12}$, and the average expected utility is $U_{1}$, as equations Equation 1, Equation 2 and Equation 3, then:

$$\begin{array}{c} U_{11}=yz\left(aF_{1}+R_{12}+R_{13}-C_{12}-C_{13}\right)+y\left(1-z\right)\left(R_{12}-C_{12}\right)+\left(1-y\right)z\left(bS+R_{13}-C_{13}\right)+\\ \left(1-y\right)\left(1-z\right)\left(bS+aF_{1}\right)=2yzaF_{1}+yR_{12}-yC_{12}+zR_{13}-zC_{13}+bS+aF_{1}-ybS-yaF_{1}-zaF_{1}\left(1\right) \end{array}$$

(2) $$\begin{array}{c} U_{12}=yz\left(-P_{1}\right)+y\left(1-z\right)\left(-P_{1}\right)+\left(1-y\right)z\left(-P_{1}\right)+\left(1-y\right)\left(1-z\right)\left(-p_{1}\right)=-P_{1} \end{array}$$

(3) $$\begin{array}{c} U_{1}=xU_{11}+\left(1-x\right)U_{12} \end{array}$$

The replicated dynamic equation of local government G(x) is Equation (4):

(4) $$G\left(x\right)=\frac{dx}{dt}=x\left(U_{11}-U_{1}\right)=x\left(1-x\right)\left(U_{11}-U_{12}\right)=x\left(1-x\right)\left(2yzaF_{1}+yR_{12}-yC_{12}+zR_{13}-zC_{13}+bS+aF_{1}-ybS-yaF_{1}-zaF_{1}+P_{1}\right)$$

Assuming that the expected utility of the support strategy of the capital is $U_{21}$, the expected utility of the non-support strategy of the capital is $U_{22}$, and the average expected utility is $U_{2}$, asEquation 5, Equation 6 and Equation 7, then:

$$U_{21}=xz\left(R_{22}+{aF}_{2}+C_{12}-C_{2}-C_{21}\right)+x\left(1-z\right)\left(R_{22}+{aF}_{2}+C_{12}-C_{2}-C_{21}\right)+\left(1-x\right)z\left(R_{22}+{aF}_{2}+C_{12}-C_{2}-C_{21}\right)+\left(1-x\right)\left(1-z\right)\left(R_{22}+{aF}_{2}+C_{12}-C_{2}-C_{21}\right)=xC_{12}+R_{22}+aF_{2}-C_{21}\left(5\right)$$

(6) $$U_{22}=xz\left(R_{21}-M_{1}-bS\right)+x\left(1-z\right)\left(R_{21}-M_{1}-bS\right)+\left(1-x\right)z\left(R_{21}-M_{1}\right)+\left(1-x\right)\left(1-z\right)\left(R_{21}-M_{1}\right)=-xbS+R_{21}-M_{1}$$

(7) $$\begin{array}{c} U_{2}=yU_{21}+\left(1-y\right)U_{22} \end{array}$$

The replicated dynamic equation of capital S(y) is Equation (8):

(8) $$S\left(y\right)=\frac{dy}{dt}=y\left(U_{21}-U_{2}\right)=y\left(1-y\right)\left(U_{21}-U_{22}\right)=y\left(1-y\right)\left(xC_{12}+R_{22}+aF_{2}-C_{21}+xbS-R_{21}+M_{1}\right)$$

Assuming that the expected utility of the positivity strategy of the people is $U_{31}$, the expected utility of the non-positivity strategy of the people is $U_{32}$, and the average expected utility is $U_{3}$, as Equation 9, Equation 10 and Equation 11, then:

$$U_{31}=xy\left(C_{13}+R_{32}+aF_{3}-C_{3}\right)+x\left(1-y\right)\left(C_{13}+R_{32}+aF_{3}-C_{3}\right)+\left(1-x\right)y\left(R_{32}+aF_{3}-C_{3}\right)+\left(1-x\right)\left(1-y\right)\left(R_{32}+aF_{3}-C_{3}\right)=xC_{13}+R_{32}+aF_{3}-C_{3}\left(9\right)$$

(10) $$\begin{array}{c} U_{32}=xyR_{31}+x\left(1-y\right)R_{31}+\left(1-x\right)yR_{31}+\left(1-x\right)\left(1-y\right)R_{31}=R_{31} \end{array}$$

(11) $$\begin{array}{c} U_{3}=zU_{31}+\left(1-z\right)U_{32} \end{array}$$

The replicated dynamic equation of capital N(z) isEquation 12:

(12) $$N\left(z\right)=\frac{dz}{dt}=z\left(U_{31}-U_{3}\right)=z\left(1-z\right)\left(U_{31}-U_{32}\right)=z\left(1-z\right)\left(xC_{13}+R_{32}+aF_{3}-C_{3}-R_{31}\right)$$

To further explore the concept of evolutionarily stable points in the tri-partite game, let's consider the following set of simultaneous equations Equation 13:

(13) $$\begin{array}{c} G\left(x\right)=\frac{dx}{dt}=x\left(U_{11}-U_{1}\right)=x\left(1-x\right)\left(U_{11}-U_{12}\right)=x\left(1-x\right)\left(2yzaF_{1}+yR_{12}-yC_{12}+zR_{13}-zC_{13}+bS+aF_{1}-ybS-yaF_{1}-zaF_{1}+P_{1}\right)=0\\ S\left(y\right)=\frac{dy}{dt}=y\left(U_{21}-U_{2}\right)=y\left(1-y\right)\left(U_{21}-U_{22}\right)=y\left(1-y\right)\left(xC_{12}+R_{22}+aF_{2}-C_{21}+xbS-R_{21}+M_{1}\right)=0\\ N\left(z\right)=\frac{dz}{dt}=z\left(U_{31}-U_{3}\right)=z\left(1-z\right)\left(U_{31}-U_{32}\right)=z\left(1-z\right)\left(xC_{13}+R_{32}+aF_{3}-C_{3}-R_{31}\right)=0 \end{array}$$

From the above simultaneous equations, the evolutionary stable points of local government, capital, and people are obtained: $E_{1}\left(0,0,0\right)$, $E_{2}\left(0,0,1\right)$, $E_{3}\left(0,1,0\right)$, $E_{4}\left(0,1,1\right)$, $E_{5}\left(1,0,0\right)$, $E_{6}\left(1,0,1\right)$, $E_{7}\left(1,1,0\right)$, $E_{8}\left(1,1,1\right)$ and $E_{9}\left(x^{*},y^{*},z^{*}\right)$, where $E_{9}\left(x^{*},y^{*},z^{*}\right)$ satisfies the following simultaneous Equation (14):

(14) $$\left\{ \begin{array}{c} 2yzaF_{1}+yR_{12}-yC_{12}+zR_{13}-zC_{13}+bS+aF_{1}-ybS-yaF_{1}-zaF_{1}+P_{1}=0\\ xC_{12}+R_{22}+aF_{2}-C_{21}+xbS-R_{21}+M_{1}=0\\ xC_{13}+R_{32}+aF_{3}-C_{3}-R_{31}=0 \end{array}\right.$$

## 4 Evolutionary stability analysis

### 4.1 Asymptotic stability analysis of the three parties

#### 4.1.1 Asymptotic stability analysis of local government

**Fig. 2:**
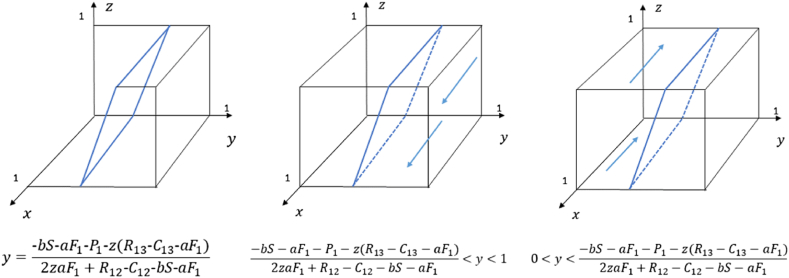
Phase diagram of strategy evolution of the local government.

According to the stability theorem of the replicated dynamical equation, if the following two conditions G(x)=0 and $G^{\prime }\left(x\right)<0$ are satisfied, x is the evolutionary stable point, where $G^{\prime }\left(x\right)=\left(1-2x\right)\left[2yzaF_{1}+\left(R_{12}-C_{12}-bS-aF_{1}\right)y+\left(R_{13}-C_{13}-aF_{1}\right)z+bS+aF_{1}+P_{1}\right]$. The strategy evolution diagram of the local government is shown in Fig. 2.

Let G(x)=0, then x=0, x=1, and $y=\frac{-bS-aF_{1}-P_{1}-z\left(R_{13}-C_{13}-aF_{1}\right)}{2zaF_{1}+R_{12}-C_{12}-bS-aF_{1}}$.

1) When $y=\frac{-bS-aF_{1}-P_{1}-z\left(R_{13}-C_{13}-aF_{1}\right)}{2zaF_{1}+R_{12}-C_{12}-bS-aF_{1}}$, G(x)=0 is always satisfied. The stability remains constant regardless of the value x takes within the given interval, ensuring that the local government's strategy remains unchanged over time.
2) When $0<y<\frac{-bS-aF_{1}-P_{1}-z\left(R_{13}-C_{13}-aF_{1}\right)}{2zaF_{1}+R_{12}-C_{12}-bS-aF_{1}}$, $2yzaF_{1}+\left(R_{12}-C_{12}-bS-aF_{1}\right)y+\left(R_{13}-C_{13}-aF_{1}\right)z+bS+aF_{1}+P_{1}<0$ holds. By substituting x = 0 and x = 1 into G′(x), and obtaining G′(0)<0, G′(1)>0. It follows that x = 0 represents the evolutionary stable point. This implies that as the probability of capital choosing to support industrial upgrading steadily decreases below a certain threshold, the probability of the government choosing the incentive strategy gradually decreases as well. Eventually, the government will choose the non-incentive strategy.
3) When $\frac{-bS-aF_{1}-P_{1}-z\left(R_{13}-C_{13}-aF_{1}\right)}{2zaF_{1}+R_{12}-C_{12}-bS-aF_{1}}<y<1$, $2yzaF_{1}+\left(R_{12}-C_{12}-bS-aF_{1}\right)y+\left(R_{13}-C_{13}-aF_{1}\right)z+bS+aF_{1}+P_{1}>0$ holds. Bysubstituting x = 0 and x = 1 into G′(x), and obtaining G′(0)>0, G′(1)<0. It follows that x = 1 represents the evolutionary stable point. This means that as the probability that capital will support the upgrading of the industrial structure exceeds a certain threshold and continues to increase, the probability that the government will opt for the incentive strategy will correspondingly increase. Ultimately, the government will choose the incentive strategy.

#### 4.1.2 Asymptotic stability analysis of capital

**Fig. 3:**
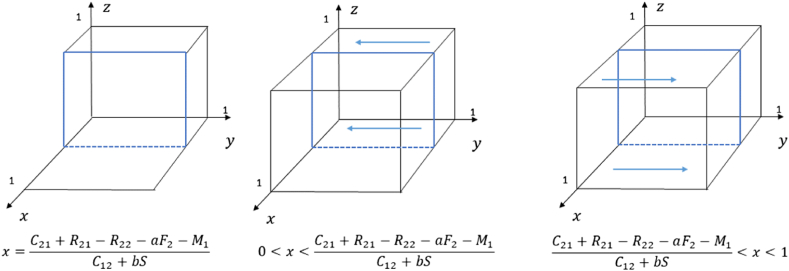
Phase diagram of strategy evolution of the capital.

According to the stability theorem of the replicated dynamical equation, if the following two conditions S(y)=0 and $S^{\prime }\left(y\right)<0$ are satisfied, y is the evolutionary stable point, where $S^{\prime }\left(y\right)=\left(1-2y\right)\left[\left(C_{12}+bS\right)x+R_{22}+aF_{2}-C_{21}-R_{21}+M_{1})\right]$. The strategy evolution diagram of the capital is shown in Fig. 3.

$C_{12}+bS>0$ is satisfied. Let S(y)=0, then y=0, y=1, and $x=\frac{C_{21}+R_{21}-R_{22}-aF_{2}-M_{1}}{C_{12}+bS}$.

1) When $x=\frac{C_{21}+R_{21}-R_{22}-aF_{2}-M_{1}}{C_{12}+bS}$, S(y)=0 is always satisfied. The stability remains constant regardless of the value y takes within the given interval, ensuring that the capital's strategy remains unchanged over time.
2) When $0<x<\frac{C_{21}+R_{21}-R_{22}-aF_{2}-M_{1}}{C_{12}+bS}$, $\left(C_{12}+bS\right)x+R_{22}+aF_{2}-C_{21}-R_{21}+M_{1})<0$ holds. Bysubstituting y = 0 and y = 1 into S ′ (y), and obtainingS ′ (0)<0, S ′ (1)>0. It follows that y = 0 represents the point of evolutionary stability. This means that as the probability of the local government choosing the incentive strategy falls below a certain threshold and continues to fall, the probability of capital choosing the support strategy gradually decreases. Eventually, capital will choose the non-support strategy.
3) When $\frac{C_{21}+R_{21}-R_{22}-aF_{2}-M_{1}}{C_{12}+bS}<x<1$, $\left(C_{12}+bS\right)x+R_{22}+aF_{2}-C_{21}-R_{21}+M_{1})>0$ holds. By substituting y = 0 and y = 1 into S′(y), and obtaining S′(0)>0, S′(1)<0. It follows that y = 1 represents the point of evolutionary stable point. This suggests that once the probability of the local government choosing the incentive strategy surpasses a specific threshold and continues to rise, capital will increasingly choose the support strategy. Eventually, capital will choose the support strategy.

#### 4.1.3 Asymptotic stability analysis of people

**Fig. 4:**
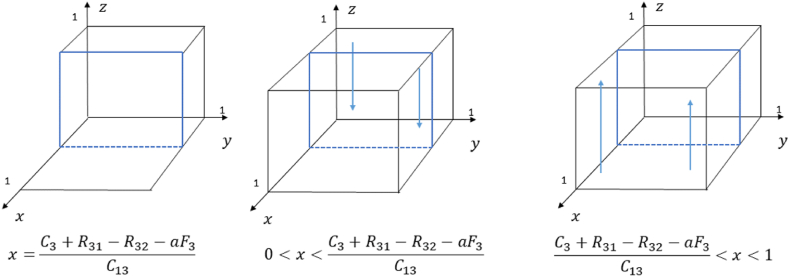
Phase diagram of strategy evolution of the people.

According to the stability theorem of the replicated dynamical equation, if the following two conditions N(z)=0 and $N^{\prime }\left(z\right)<0$ are satisfied, z is the evolutionary stable point, where $N^{\prime }\left(z\right)=\left(1-2z\right)\left(xC_{13}+R_{32}+aF_{3}-C_{3}-R_{31}\right)$. The strategy evolution diagram of the people is shown in Fig. 4.

Let N(z)=0, then z=0, z=1, and $x=\frac{C_{3}+R_{31}-R_{32}-aF_{3}}{C_{13}}$.

1) When $x=\frac{C_{3}+R_{31}-R_{32}-aF_{3}}{C_{13}}$, N(z)=0 is always satisfied. The stability remains constant regardless of the value z takes within the given interval, ensuring that the people's strategy remains unchanged over time.
2) When $0<x<\frac{C_{3}+R_{31}-R_{32}-aF_{3}}{C_{13}}$, $xC_{13}+R_{32}+aF_{3}-C_{3}-R_{31}<0$ holds. By substituting z = 0 and z = 1 into N′(z), and obtaining N′(0)<0, N′(1)>0. It follows that z = 0 represents the evolutionary stable point. This indicates that as the probability of the local government choosing an incentive strategy falls below a certain threshold and keeps falling, the probability of the people choosing a positivity strategy gradually decreases. Finally, people will choose the non-positive strategy.
3) When $\frac{C_{3}+R_{31}-R_{32}-aF_{3}}{C_{13}}<x<1$, $xC_{13}+R_{32}+aF_{3}-C_{3}-R_{31}>0$ holds. By substituting z = 0 and z = 1 into N′(z), and obtaining N′(0)>0, N′(1)<0. It follows that z = 1 represents the evolutionary stable point. This suggests that the probability of the local government choosing the incentive strategy rises above a certain threshold and continues to rise, the probability of the people choosing the positivity strategy increases accordingly. Eventually, the people choose the positivity strategy.

### 4.2 Stability analysis of tripartite evolutionary game system

According to Friedman [43], The determination of the Evolutionarily Stable Strategy (ESS) within the game can be achieved by analyzing the local stability of the Jacobian matrix. In this paper, we present the Jacobian matrix associated with the evolutionary game model of industrial structure upgrading as follows(Equation 15):(15)
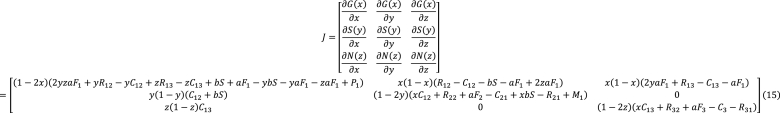
In the asymmetric evolutionary game, it is appropriate to focus merely on the stability of pure strategy equilibria. Thus, we will discuss the stability of eight equilibrium points: $E_{1}\left(0,0,0\right)$, $E_{2}\left(0,0,1\right)$, $E_{3}\left(0,1,0\right)$, $E_{4}\left(0,1,1\right)$, $E_{5}\left(1,0,0\right)$, $E_{6}\left(1,0,1\right)$, $E_{7}\left(1,1,0\right)$, and $E_{8}\left(1,1,1\right)$. The hybrid strategy equilibrium $E_{9}$ is not considered an Evolutionarily Stable Strategy (ESS). According to Lyapunov's stability theory [[43], [44], [45]], an equilibrium point qualifies as an ESS only if all eigenvalues of the Jacobian matrix are negative. If the sign of all eigenvalues of the Jacobian matrix can be determined, and there is at least one positive eigenvalue, the equilibrium point is considered unstable; otherwise, it is classified as a saddle point. The eigenvalues of the Jacobian matrix can be calculated by substitution of the eight equilibrium points into the matrix, as shown in Table 3.

**Table 3:** Eigenvalues of the Jacobian matrix.

Equilibrium points	Eigenvalue 1	Eigenvalue 2	Eigenvalue 3	Stability	Stable conditions
$E_{1}\left(0,0,0\right)$	$bS+aF_{1}+P_{1}$	$R_{22}+aF_{2}-C_{21}-R_{21}+M_{1}$	$R_{32}+aF_{3}-C_{3}-R_{31}$	Unstable	
$E_{2}\left(0,0,1\right)$	$R_{13}-C_{13}+bS+P_{1}$	$R_{22}+aF_{2}-C_{21}-R_{21}+M_{1}$	$-R_{32}-aF_{3}+C_{3}+R_{31}$	Unstable	
$E_{3}\left(0,1,0\right)$	$R_{12}-C_{12}+P_{1}$	$-R_{22}-aF_{2}+C_{21}+R_{21}+M_{1}$	$R_{32}+aF_{3}-C_{3}-R_{31}$	ESS	$C_{12}>R_{12}+P_{1}$ $R_{22}+aF_{2}>C_{21}+R_{21}+M_{1}$ $C_{3}+R_{31}>R_{32}+aF_{3}$
$E_{4}\left(0,1,1\right)$	$aF_{1}+R_{12}-C_{12}+R_{13}-C_{13}+P_{1}$	$-R_{22}-aF_{2}+C_{21}+R_{21}-M_{1}$	$-R_{32}-aF_{3}+C_{3}+R_{31}$	ESS	$C_{12}+C_{13}>aF_{1}+R_{12}+R_{13}+P_{1}$ $R_{22}+aF_{2}+M_{1}>C_{21}+R_{21}$ $R_{32}+aF_{3}>C_{3}+R_{31}$
$E_{5}\left(1,0,0\right)$	$-bS-aF_{1}-P_{1}$	$C_{12}+R_{22}+aF_{2}-C_{21}+bS-R_{21}+M_{1}$	$C_{13}+R_{32}+{aF}_{3}-C_{3}-R_{31}$	ESS	$C_{21}+R_{21}>C_{12}+R_{22}+aF_{2}+bS+M_{1}$ $C_{3}+R_{31}>C_{13}+R_{32}+aF_{3}$
$E_{6}\left(1,0,1\right)$	$-R_{13}+C_{13}-bS-P_{1}$	$C_{12}+R_{22}+aF_{2}-C_{21}+bS-R_{21}+M_{1}$	$-C_{13}-R_{32}-{aF}_{3}+C_{3}+R_{31}$	ESS	$R_{13}+bS+P_{1}>C_{13}$ $C_{21}+R_{21}>C_{12}+R_{22}+aF_{2}+bS+M_{1}$ $C_{13}+R_{32}+aF_{3}>C_{3}+R_{31}$
$E_{7}\left(1,1,0\right)$	$-R_{12}+C_{12}-P_{1}$	$-C_{12}-R_{22}-aF_{2}+C_{21}-bS+R_{21}-M_{1}$	$C_{13}+R_{32}+{aF}_{3}-C_{3}-R_{31}$	ESS	$R_{12}+P_{1}>C_{12}$ $C_{12}+R_{22}+aF_{2}+M_{1}>C_{21}+R_{21}$ $C_{3}+R_{31}>C_{13}+R_{32}+aF_{3}$
$E_{8}\left(1,1,1\right)$	$-aF_{1}-R_{12}+C_{12}-R_{13}+C_{13}-P_{1}$	$-C_{12}-R_{22}-aF_{2}+C_{21}-bS+R_{21}-M_{1}$	$-C_{13}-R_{32}-{aF}_{3}+C_{3}+R_{31}$	ESS	$aF_{1}+R_{12}+R_{13}+P_{1}>C_{12}+C_{13}$ $C_{12}+R_{22}+aF_{2}+bS+M_{1}>C_{21}+R_{21}$ $C_{13}+R_{32}+aF_{3}>C_{3}+R_{31}$

#### 4.2.1 Scenario 1：

When $C_{12}>R_{12}+P_{1}$, $R_{22}+aF_{2}>C_{21}+R_{21}+M_{1}$, and $C_{3}+R_{31}<R_{32}+aF_{3}$, the negative values of all eigenvalues in the Jacobian matrix for the evolutionary sta-ble point $E_{3}\left(0,1,0\right)$ indicate that $E_{3}\left(0,1,0\right)$ is an ESS. This suggests that i) when the government subsidies for capital is higher than the sum of the government benefits from capital and the government punishment from higher levels of government; ii) when the sum of the current income of the capital from cultural tourism industry and future income of capital, is higher than the sum of transformation costs paid by capital, the current income of the capital from resource-based industry, carbon trading fee; iii) when the sum of the current income of the people in the cultural tourism industry, future income of people is higher than the sum of the cost to the people for changing, current people's income in resource-based industries, (Non-incentive, Support, Non-positivity) is the ESS.

#### 4.2.2 Scenario 2：

When $C_{12}+C_{13}>aF_{1}+R_{12}+R_{13}+P_{1}$, $R_{22}+aF_{2}+M_{1}>C_{21}+R_{21}$, and $R_{32}+aF_{3}>C_{3}+R_{31}$, the negative values of all eigenvalues in the Jacobian matrix for the evolutionary stable point $E_{4}\left(0,1,1\right)$ indicate that $E_{4}\left(0,1,1\right)$ is an ESS. This suggests that i)when the sum of government subsidies for capital, government subsidies for people is higher than the sum of future benefits for local government, government benefits from capital, government benefits from the people, government punishment from higher government; ii) when the sum of the current income of the capital from cultural tourism industry, future income of capital, carbon trading fees, is higher than the sum of transformation costs paid by capital, the current income of the capital from resource-based industry; iii) when the sum of current income of people from cultural tourism industry, future income of people, is higher than the sum of cost to the people for changing, current income of people from resource-based industry, (Non-incentive, Support, Positivity) is the ESS.

#### 4.2.3 Scenario 3：

When $C_{21}+R_{21}>C_{12}+R_{22}+aF_{2}+bS+M_{1}$ and $C_{3}+R_{31}>C_{13}+R_{32}+aF_{3}$, the negative values of all eigenvalues in the Jacobian matrix for the evolutionary stable point $E_{5}\left(1,0,0\right)$ indicate that $E_{5}\left(1,0,0\right)$ is an ESS. This suggests that i) when the sum of transformation costs paid by capital, the current income of the capital from resource-based industry is higher than the sum of government subsidies for capital, the current income of the capital from cultural tourism industry, future income of capital, carbon tax, carbon trading fees; ii) when the sum of cost to the people for changing, current income of people from resource-based industry, is higher than the sum of government subsidies for people, current income of people from cultural tourism industry, (Incentive, Non-support, Non-positivity) is the ESS.

#### 4.2.4 Scenario 4：

When $R_{13}+bS+P_{1}>C_{13}$, $C_{21}+R_{21}>C_{12}+R_{22}+aF_{2}+bS+M_{1}$, and $C_{13}+R_{32}+aF_{3}>C_{3}+R_{31}$, the negative values of all eigenvalues in the Jacobian matrix for the evolutionary stable point $E_{6}\left(1,0,1\right)$ indicate that $E_{6}\left(1,0,1\right)$ is an ESS. This suggests that i) when the sum of government benefits from the people, carbon tax, government punishment from higher government is higher than government subsidies for people; ii) when the sum of transformation costs paid by capital, the current income of the capital from resource-based industry is higher than the sum of government subsidies for capital, the current income of the capital from cultural tourism industry, future income of capital, carbon tax, carbon trading fees; iii) when the sum of government subsidies for people, current income of people from cultural tourism industry, future income of people is higher than the sum of cost to the people for changing, current income of people from resource-based industry, (Incentive, Non-support, Positivity) is the ESS.

#### 4.2.5 Scenario 5：

When $R_{12}+P_{1}>C_{12}$, $C_{12}+R_{22}+aF_{2}+M_{1}>C_{21}+R_{21}$, and $C_{3}+R_{31}>C_{13}+R_{32}+aF_{3}$, the negative values of all eigenvalues in the Jacobian matrix for the evolutionary stable point $E_{7}\left(1,1,0\right)$ indicate that $E_{7}\left(1,1,0\right)$ is an ESS. This suggests that i) when the sum of government benefits from capital, government punishment from higher government is higher than the sum of government subsidies for capital; ii) when the sum of government subsidies for capital, the current income of the capital from cultural tourism industry, future income of capital, carbon trading fees, is higher than the sum of transformation costs paid by capital, the current income of the capital from resource-based industry; iii) when the sum of cost to the people for changing, current income of people from resource-based industry is higher than the sum of government subsidies for people, current income of people from cultural tourism industry, future income of people, (Incentive, Support, Non-positivity) is the ESS.

#### 4.2.6 Scenario 6：

When $aF_{1}+R_{12}+R_{13}+P_{1}>C_{12}+C_{13}$, $C_{12}+R_{22}+aF_{2}+bS+M_{1}>C_{21}+R_{21}$, and $C_{13}+R_{32}+aF_{3}>C_{3}+R_{31}$, the negative values of all eigenvalues in the Jacobian matrix for the evolutionary stable point $E_{8}\left(1,1,1\right)$ indicate that $E_{8}\left(1,1,1\right)$ is an ESS. This suggests that i) when the sum of future benefits for local government, government benefits from capital, government benefits from the people, government punishment from higher government is higher than the sum of government subsidies for capital, government subsidies for people; ii) when the sum of government subsidies for capital, the current income of the capital from cultural tourism industry, future income of capital, carbon trading fees, carbon trading fees is higher than the sum of transformation costs paid by capital, the current income of the capital from resource-based industry; iii) when the sum of government subsidies for people, current income of people from cultural tourism industry, future income of people is higher than the sum of cost to the people for changing, current income of people from resource-based industry, (Incentive, Support, Positivity) is the ESS.

## 5 Numerical simulation

### 5.1 Expanding on the preceding analysis, this section investigates how different parameters impact the stability of industrial structure upgrading. To conduct a comprehensive investigation of the evolutionary dynamics among the three players, numerical simulations are utilized. Data collection and parameter settings

In this study, we used MATLAB 2020a software for conducting numerical simulations. These simulations were crucial in visualizing the iterations of the players' strategies, allowing for a quantitative analysis of their interactions. Through these simulations, we observe how the strategies change at each stage under different parameter values. In setting the initial parameters for the model, we followed two essential principles: Firstly, we reflected real-world conditions, and secondly, we took into account the logical relationships among the parameters. Subsequently, we explain the parameters for each scenario, highlighting their connections and dependencies based on these principles.

The Chinese government is strongly committed to achieving the goals of “carbon neutrality” and “carbon peaking,” making green development and industrial upgrading key strategies in various regions of China. With a high probability of success, industrial upgrading is assigned a value of 0.7, denoted by the parameter “a". This paper estimates the probability of successful industrial upgrading to be 70 %, in line with the prevailing economic development trends in Shanxi, China. This estimate is substantiated by combining the historical practices of the Shanxi provincial government with research findings on enterprise development. Similarly, the government is inclined to implement a carbon tax on resource-based enterprises, leading to the selection of 0.6 for parameter “b". At present, the mechanism for collecting a carbon tax is still relatively nascent. Nevertheless, there is a significant probability that the government will impose a carbon tax on resource-dependent enterprises. Therefore, setting the probability at 60 % is considered reasonable and prudent. Analysis of publicly available data from the Shanxi government shows that the current revenue generated by resource-based enterprises is about twice that of the cultural tourism industry [46]. As a result, parameter " $R_{21}$" is set equal to 2 multiplied by " $R_{22}$". Considering the need for significant investment in the cultural tourism industry, the cost is approximately equal to the annual revenue of the resource-based enterprises. Therefore, " $C_{21}$" can be considered approximately equal to " $R_{21}$". The annual production of coal and other resources in Shanxi is about 100 million tons, and based on carbon trading prices, " $M_{1}$" is set at 15. According to the statistics, it can be seen that cultural tourism companies offer two-thirds of the salary of resource-based companies. Therefore, according to the data comparison [47], " $R_{31}$" has a value of 60, while " $R_{32}$" has a value of 40. Since it is difficult to quantify the cost of people's adaptation to new work and lifestyle, " $C_{3}$" was set at 60, representing one year's salary in resource-based companies. Shanxi Province's historical reliance on resource-based enterprises for economic growth has had a lasting impact on the prevailing lifestyles and work patterns of its population. As a result, the transformation process is particularly challenging, warranting a rational decision to equate it with one year of people's income. The result of upgrading the industrial structure and green development of the regional economy is definitely stable to good, so in this paper, the future benefits that can be obtained are slightly larger than the current ones, so the values of " $F_{1}$", " $F_{2}$" and " $F_{3}$" are 40, 50 and 70, respectively. It is important to note that simulated systems progress in virtual time t, not in real time.

In light of the above considerations, certain parameters were rounded to improve the feasibility of performing numerical simulations. The parameter settings for various scenarios are presented in Table 4, taking into account real-world conditions and the recommendations of experts interviewed during the study.

**Table 4:** Parameters for different scenarios.

Parameters	a	$F_{1}$	$R_{12}$	$C_{12}$	b	S	$R_{13}$	$C_{13}$	$P_{1}$
Scenario 1	0.7	40	10	60	0.6	30	60	45	10
Scenario 2	0.7	40	10	60	0.6	30	60	55	10
Scenario 3	0.7	40	10	45	0.4	30	60	55	10
Scenario 4	0.7	40	10	45	0.4	30	60	55	10
Scenario 5	0.7	40	30	50	0.6	30	60	35	10
Scenario 6	0.7	40	30	50	0.6	30	60	40	10
Parameters	$R_{22}$	$F_{2}$	$C_{21}$	$R_{21}$	$M_{1}$	$R_{32}$	$F_{3}$	$C_{3}$	$R_{31}$
Scenario 1	40	80	50	60	20	40	60	60	60
Scenario 2	40	80	50	60	20	60	90	60	60
Scenario 3	40	50	80	70	15	40	40	60	60
Scenario 4	40	50	80	70	15	40	60	60	60
Scenario 5	30	50	70	70	15	40	60	60	60
Scenario 6	30	50	70	70	15	40	70	60	60

### 5.2 The dynamic evolutionary paths

Scenario analysis plays a crucial role in exploring the varying impacts of potential factor developments in different political and economic contexts. It enables the quantification of the trajectory required to achieve future goals of industrial upgrading and regional economic green development. The following text presents numerical simulations for six scenarios in the evolutionary game, providing valuable insights into the evolutionary process of each scenario.

#### 5.2.1 Scenario 1

**Fig. 5:**
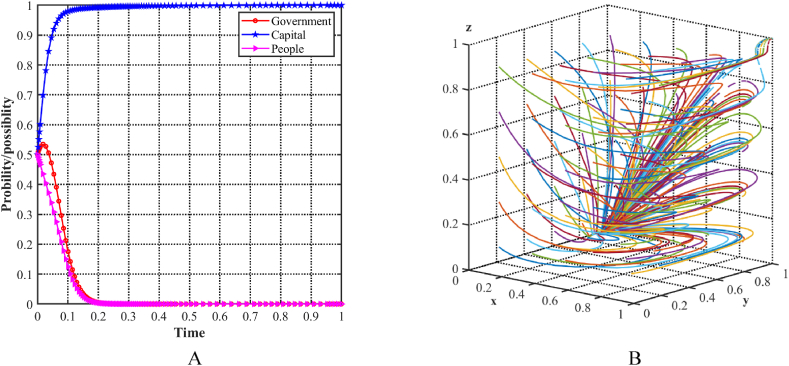
Evolutionary equilibria in the scenario 1. (A) Evolutionary trajectories of three players in the scenario 1. (B) Evolution process of the ESS.

Using the stability conditions calculated for Scenario 1, numerical simulations are run with different parameter values. Fig. 5A shows the increasing probability of the capital choosing a support strategy, while the probability of the local government and the people choosing a positive strategy decreases. The initial condition of 0.5 is assigned to all three parties. To evaluate the stability of the equilibrium point $E_{3}\left(0,1,0\right)$ in the evolutionary model, a loop is used in MATLAB software to simulate different initial strategies for the three players, generating random x, y, and z points. The decision dynamics in the three-player evolutionary game are depicted in Fig. 5B, which shows several colored lines that eventually converge to $E_{3}\left(0,1,0\right)$. Specifically, in this scenario, the local government is required to provide substantial subsidies to facilitate the development of the cultural tourism industry, while the benefits of the people adopting positive lifestyle changes are outweighed by the associated costs. Consequently, only the capital support the upgrading of the industrial structure. At this point, all parties recognize the potential for improved results by increasing the benefits to the people and reducing government expenditures.

#### 5.2.2 Scenario 2

**Fig. 6:**
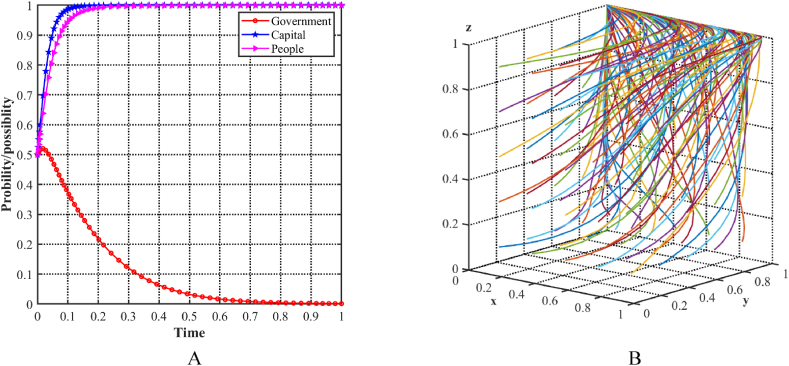
Evolutionary equilibria in the scenario 2. (A) Evolutionary trajectories of three players in the scenario 2. (B) Evolution process of the ESS.

As shown in Fig. 6A, with an initial condition of 0.5 for all three players, the probability of the capital choosing a support strategy and the people choosing a positive strategy steadily increases, while the probability of the local government choosing an incentive strategy decreases. To evaluate the stability of the equilibrium point $E_{4}\left(0,1,1\right)$ in the evolutionary model, MATLAB software is used to simulate different initial strategies cyclically by generating random x, y, and z points. Fig. 6B illustrates the decision trajectories in a three-player evolutionary game, represented by multiple colored lines that ultimately converge to $E_{4}\left(0,1,1\right)$. In this particular scenario, the local government must provide substantial subsidies to facilitate the development of the cultural tourism industry by both the capitalists and the population, while its benefits remain comparatively low. At this point, all three parties recognize that greater cooperation with the government can lead to better results.

#### 5.2.3 Scenario 3

**Fig. 7:**
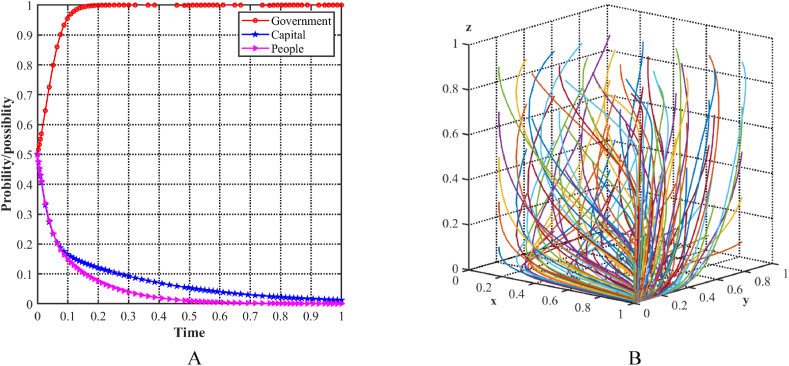
Evolutionary equilibria in the scenario 3. (A) Evolutionary trajectories of three players in the scenario 3. (B) Evolution process of the ESS.

As shown in Fig. 7A, with an initial condition of 0.5 for each of the three parties, the probability of the government choosing an incentive strategy steadily increases, while the probability of the capital and the people choosing a positive strategy decreases. To determine the stability of the equilibrium point $E_{5}\left(1,0,0\right)$ in the evolutionary model, different initial strategies for the three players are simulated by cyclically generating random x, y, and z points using MATLAB software. Fig. 7B illustrates the decision trajectories in a three-player evolutionary game, represented by multiple colored lines that ultimately converge to $E_{5}\left(1,0,0\right)$. Specifically, in this scenario, the government fails to effectively facilitate the industrial structure upgrade for both capital and the people, as the costs associated with adopting a positive strategy outweigh the benefits. At this point, recognizing the necessary conditions for promoting industrial structure upgrading and acknowledging the importance of green development may lead to more favorable results.

#### 5.2.4 Scenario 4

**Fig. 8:**
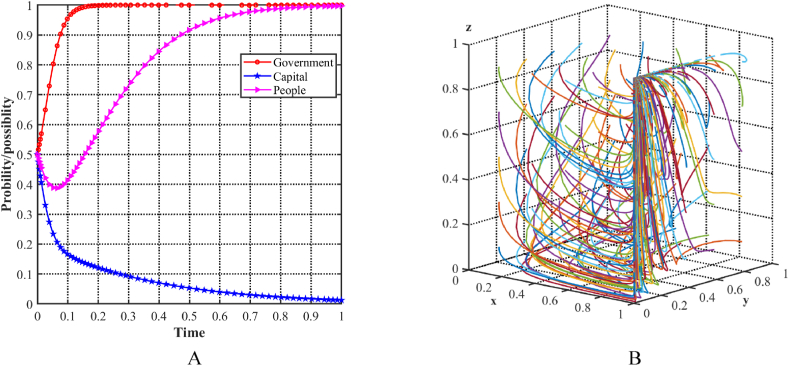
Evolutionary equilibria in the scenario 4. (A) Evolutionary trajectories of three players in the scenario 4. (B) Evolution process of the ESS.

As shown in Fig. 8A, with an initial condition of 0.5 for each of the three parties, the probability of the capital to choose a support strategy decreases, while the probability of the local government and the people choosing an aggressive strategy increases. To determine the stability of the equilibrium point $E_{6}\left(1,0,1\right)$ in the evolutionary model, different initial strategies for the three players are simulated by cyclically generating random x, y, and z points using MATLAB software. Fig. 8B illustrates the decision trajectories in a three-player evolutionary game, represented by multiple colored lines that ultimately converge to $E_{6}\left(1,0,1\right)$. Specifically, in this scenario, the local government allocates substantial resources to achieve industrial upgrading and promote green development, while the people choose an active strategy due to the potential for greater benefits. However, the capital does not support the strategy, mainly because of the significant costs associated with the transformation and the comparatively higher profits for resource-based enterprises. Consequently, the three stakeholders recognize that increasing transformation subsidies and future benefits to capital may lead to more favorable results.

#### 5.2.5 Scenario 5

**Fig. 9:**
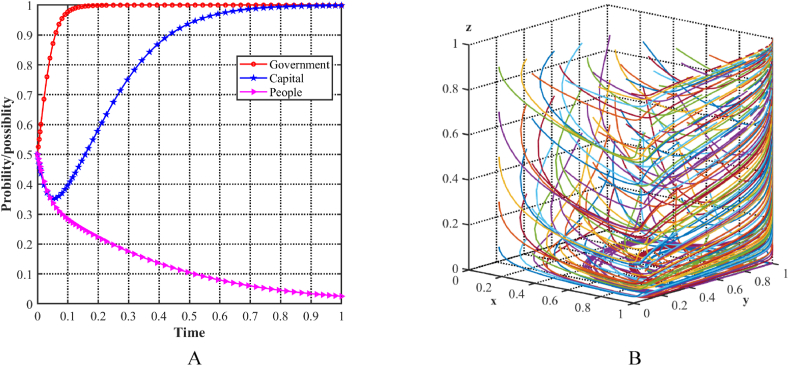
Evolutionary equilibria in the scenario 5. (A) Evolutionary trajectories of three players in the scenario 5. (B) Evolution process of the ESS.

As shown in Fig. 9A, with an initial condition of 0.5 for each of the three parties, the probability of the local government and the capital choosing a supportive strategy increases, while the probability of the people choosing a positivity strategy decreases. To evaluate the stability of the equilibrium point $E_{7}\left(1,1,0\right)$ in the evolutionary model, different initial strategies for the three players are simulated by generating random x, y, and z points in a loop using MATLAB software. Fig. 9B illustrates the decision trajectories in a three-player evolutionary game, represented by multiple colored lines that ultimately converge to $E_{7}\left(1,1,0\right)$. Specifically, in this scenario, the local government and capital establish a more favorable state, while the people choose a non-positivity strategy due to lower returns. At this point, the three parties recognize that the improvement of the industrial structure cannot neglect the participation of the people. They recognize the importance of the people's positive strategies for achieving stable and high-quality industrial structure upgrading and facilitating green development. As a result, they consider enhancing the benefits provided to the people, which could lead to better results.

#### 5.2.6 Scenario 6

**Fig. 10:**
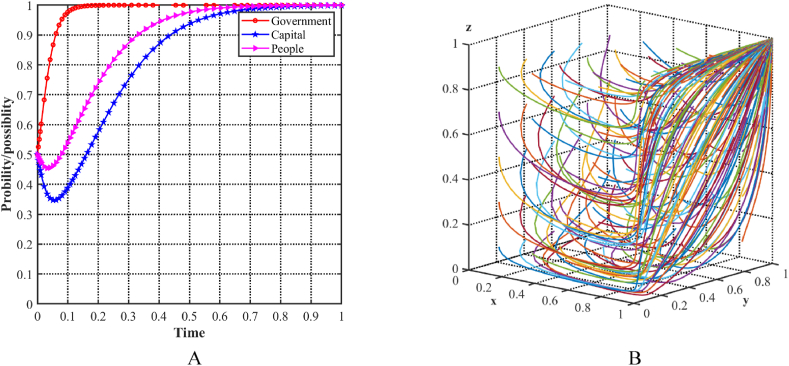
Evolutionary equilibria in the scenario 6. (A) Evolutionary trajectories of three players in the scenario 6. (B) Evolution process of the ESS.

As shown in Fig. 10A, with an initial condition of 0.5 for each of the three parties, the probability of all parties choosing a supportive strategy increases. To determine the stability of the equilibrium point $E_{8}\left(1,1,1\right)$ in the evolutionary model, different initial strategies for the three players are simulated by cyclically generating random x, y, and z points using MATLAB software. Fig. 10B illustrates the decision trajectories in a three-player evolutionary game, represented by multiple colored lines that ultimately converge to $E_{8}\left(1,1,1\right)$. Specifically, in this scenario, all three parties have positive attitudes and strategies toward upgrading the industrial structure, resulting in a more favorable equilibrium state in the three-party game. Each party demonstrates a willingness to create an optimal production and living environment conducive to green development and sustainable progress while securing its own benefits.

It is important to note that although the capital and the people eventually choose a support and positivity strategy, they initially choose a non-support and non-positivity strategy. This hesitation stems from the realization that changing industrial structure and lifestyles entails costs and requires an adjustment period, commonly referred to as the pre-change deliberation phase.

**Fig. 11:**
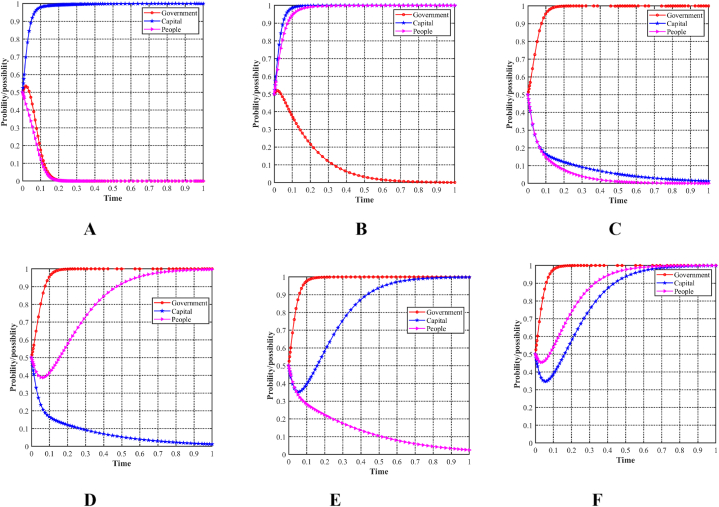
Evolutionary paths of three participants in six scenarios. (A)Scenario 1 (0,1,0). (B)Scenario 2 (0,1,1). (C)Scenario 3 (1,0,0). (D)Scenario 4 (1,0,1). (E)Scenario 5 (1,1,0). (F)Scenario 6 (1,1,1).

To enable a comprehensive comparative analysis of the six scenarios, the evolutionary trajectories of the three participants have been systematically organized and presented together in Fig. 11A, B, 11C, 11D, 11E and 11FF). This arrangement enhances the accessibility for comparing the evolutionary trajectories across the different scenarios.

### 5.3 Sensitivity analysis of key parameters

Sensitivity analysis is performed to evaluate parameters that significantly affect the system and thus determine its sensitivity to variations in these parameters. This study utilizes numerical simulations to assess the impact of crucial parameters on the evolutionary outcomes and trajectories of the tripartite Evolutionarily Stable Strategy (ESS). Parameters namely the probability of success in upgrading industrial structures (a), probability of the government collecting a carbon tax (b), government subsidies for capital $\left(C_{12}\right)$, government subsidies for people $\left(C_{13}\right)$, the cost to the people for changing the way they live and work $\left(C_{3}\right)$, transformation costs paid by capital when choosing support strategy $\left(C_{21}\right)$, future income of people after industrial structure upgrade $\left(F_{3}\right)$, Current income of resource-based industries operated by the capital $\left(R_{21}\right)$, the current income of the capital to operate the cultural tourism industry $\left(R_{22}\right)$, and the initial willingness $\left(x_{0},y_{0},z_{0}\right)$ are selected. The initial parameters are selected to meet the conditions specified in $E_{8}\left(1,1,1\right)$. Consequently, the following discussion focuses on analyzing the impact of these parameters on the evolutionary results and trajectories of the local government's incentive strategy, the capital's support strategy, and the people's positivity strategy.

#### 5.3.1 The dynamic impact of the parameter a

**Fig. 12:**
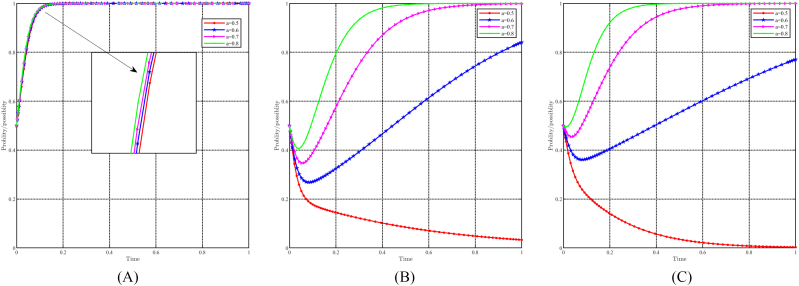
Impact of a on evolutionary results and trajectories. (A) Evolution of local government. (B) Evolution of capital. (C) Evolution of people.

When “a” takes values of 0.5, 0.6, 0.7, and 0.8, with all other parameters held constant, the effect of “a" on evolutionary results and paths is shown in Fig. 12A, B and 12C. Generally speaking, the evolutionary stable point $E_{8}\left(1,1,1\right)$ will evolve into $E_{5}\left(1,0,0\right)$ as a decreases. For local governments, variations in parameter “a" do not significantly affect their strategy choices. They consistently choose incentive strategies and higher values of “a" to speed up their decision-making process. On the other hand, both capital and the people are significantly affected by changes in “a". A lower value of “a" causes capital to choose a non-support strategy, while a lower value of “a" causes people to choose a non-positivity strategy. Notably, changes in “a" have a more pronounced effect on the people compared to the capital. In other words, for identical values of “a", the people consistently reach a stable strategy faster.

#### 5.3.2 The dynamic impact of the parameter b

**Fig. 13:**
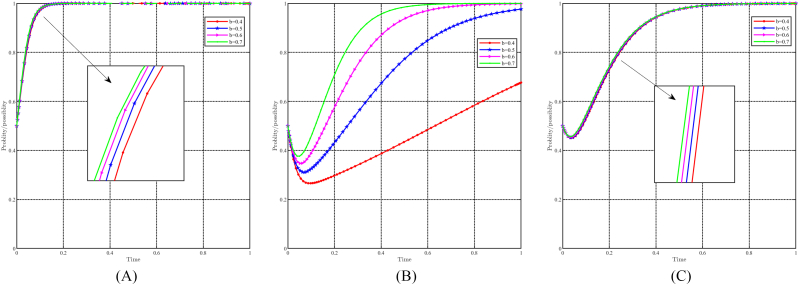
Impact of b on evolutionary results and trajectories. (A) Evolution of local government. (B) Evolution of capital. (C) Evolution of people.

When b takes values of 0.4, 0.5, 0.6, and 0.7, with all other parameters held constant, the effect of “b" on evolutionary results and paths is shown in Fig. 13A, B and 13C. The variation in the b-value does not have a greater impact on the local government and the people. However, a higher b-value leads to faster attainment of stabilization strategies by both the government and the population. In the case of capital, a decrease in the b-value undermines their commitment to choosing a support strategy. A lower b-value implies lower costs for capital that choose to non-support strategy, leading to increased hesitancy in their decision-making process.

#### 5.3.3 The dynamic impact of the parameter $C_{3}$

**Fig. 14:**
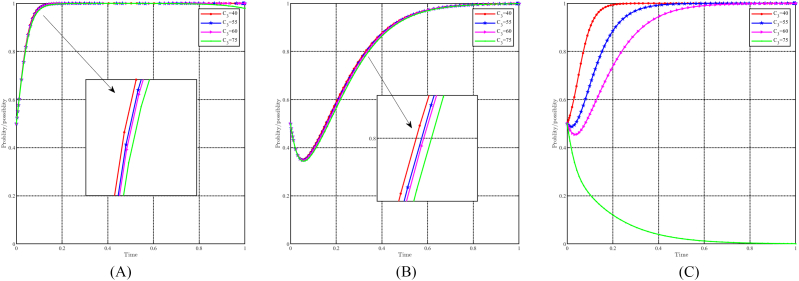
Impact of $C_{3}$ on evolutionary results and trajectories. (A) Evolution of local government. (B) Evolution of capital. (C) Evolution of people.

When $C_{3}$ takes values of 40, 55, 60, and 75, with all other parameters held constant, the effect of " $C_{3}$" on evolutionary results and paths is shown in Fig. 14A, B and 14C. In general, it can be expected that the evolutionarily stable point $E_{8}\left(1,1,1\right)$ will evolve into $E_{7}\left(1,1,0\right)$ as $C_{3}$ increases. The impact of changes in $C_{3}$ on strategy choice is minimal for local governments and funders. The only discernible effect is that a higher $C_{3}$ value leads to a slower evolution in their decision-making process. Conversely, for the people, C3 has a negative impact on their choice of positivity strategy. When $C_{3}\leq 60$, the people choose positivity strategy, but higher values of $C_{3}$ hinder their evolution. When $C_{3}\geq 75$, people tend to choose the non-positivity strategy. In particular, the costs associated with changing lifestyles do not affect the decisions of the government and capital, while higher costs tend to bias the people toward keeping the status quo.

#### 5.3.4 The dynamic impact of the parameter $C_{12}$

**Fig. 15:**
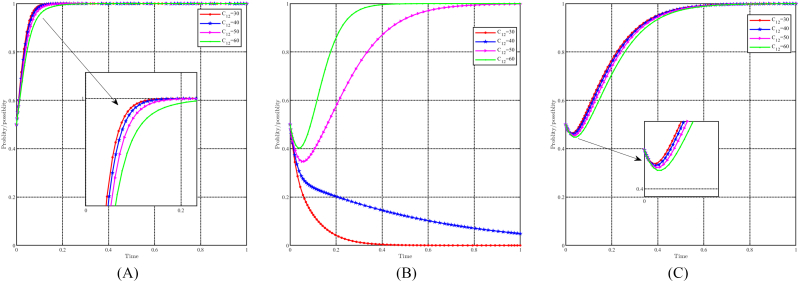
Impact of $C_{12}$ on evolutionary results and trajectories. (A) Evolution of local government. (B) Evolution of capital. (C) Evolution of people.

When $C_{12}$ takes values of 30, 40, 50, and 60, with all other parameters held constant, the effect of " $C_{12}$" on evolutionary results and paths is shown in Fig. 15A, B and 15C. In general, it can be expected that the evolutionarily stable point $E_{8}\left(1,1,1\right)$ will evolve into $E_{6}\left(1,0,1\right)$ as $C_{12}$ decreases. Changes in $C_{12}$ have a modest effect on the strategic choices of the government and the public, with a higher $C_{12}$ leading to a slower evolution. In particular, $C_{12}$ has a stronger effect on the government than on the public. Conversely, for capital, variations in $C_{12}$ significantly influence their propensity to choose support strategies. When $C_{12}\leq 40$, capital chooses a non-support strategy, and smaller $C_{12}$ values result in faster evolution. Conversely, when $C_{12}\geq 50$, capital chooses to support strategy, and higher $C_{12}$ values correspond to a faster rate of evolution. It is worth noting that $C_{12}$ represents the government's subsidy to the capital's support strategy, which influences the capital's strategic choices to some extent, but not the people's strategic choices. The government, aiming at green regional economic development, considers $C_{12}$ as a cost it is willing to bear, so changes in $C_{12}$ only marginally affect its strategy choice.

#### 5.3.5 The dynamic impact of the parameter $C_{13}$

**Fig. 16:**
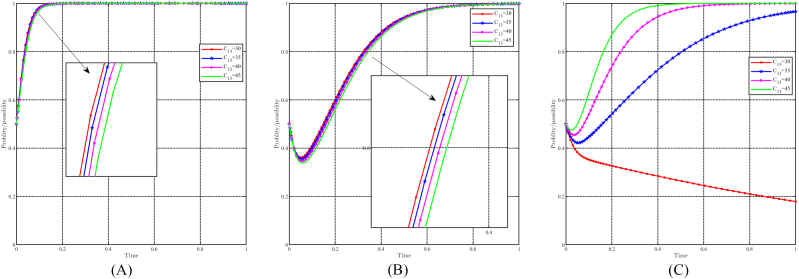
Impact of $C_{13}$ on evolutionary results and trajectories. (A) Evolution of local government. (B) Evolution of capital. (C) Evolution of people.

When $C_{13}$ takes values of 30, 35, 40, and 45, with all other parameters held constant, the effect of " $C_{13}$" on evolutionary results and paths is shown in Fig. 16A, B and 16C. In general, it can be expected that the evolutionarily stable point $E_{8}\left(1,1,1\right)$ will evolve into $E_{7}\left(1,1,0\right)$ as $C_{13}$ decreases. Changes in c13 have minimal impact on the government and capital, with a lower $C_{13}$ speeding up their rate of evolution. Conversely, changes in $C_{13}$ have a positive effect on people's choice of positivity strategy. Higher $C_{13}$ values increase the probability that the people choose positivity strategy and accelerate their evolution, while lower $C_{13}$ values tend to steer the people towards non-positivity strategies. Since $C_{13}$ represents the government's subsidy to the people, its impact on the government's strategy choice is relatively insignificant due to the government's goal of achieving green development and the relatively modest subsidy provided to the people. However, subsidies are of paramount importance to the well-being of the people, and thus the value of $C_{13}$ significantly influences the strategy choices of the people, which affects their quality of life.

#### 5.3.6 The dynamic impact of the parameter $C_{21}$

**Fig. 17:**
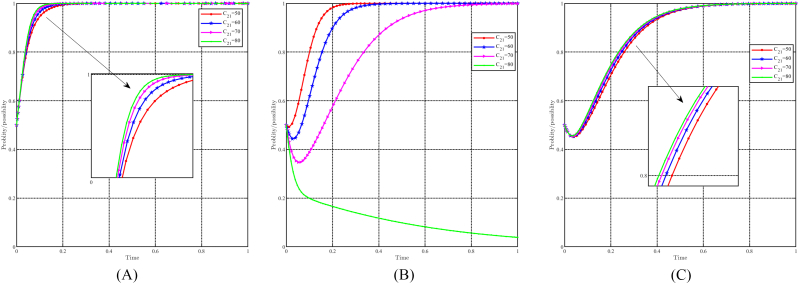
Impact of $C_{21}$ on evolutionary results and trajectories. (A) Evolution of local government. (B) Evolution of capital. (C) Evolution of people.

When $C_{21}$ takes values of 50, 60, 70, and 80, with all other parameters held constant, the effect of " $C_{21}$" on evolutionary results and paths is shown in Fig. 17A, B and 17C. In general, it can be expected that the evolutionarily stable point $E_{8}\left(1,1,1\right)$ will evolve into $E_{6}\left(1,0,1\right)$ as $C_{21}$ increases. Changes in $C_{21}$ have minimal impact on the government and the people, resulting in a longer time to reach their evolutionary stable point when $C_{21}$ is smaller. For capital, however, changes in $C_{21}$ negatively affect their choice of support strategy. A smaller $C_{21}$ value increases the probability of choosing a support strategy and accelerates its evolution. Conversely, when $C_{21}\geq 80$, the capital chooses a non-support strategy. Notably, $C_{21}$ represents the transition costs incurred by the capital in choosing the support strategy, and thus its impact on the government and the people is not great. Within a certain range, $C_{21}$ causes hesitation among capitalists and may even cause them to revert to the non-support strategy due to the diminished benefits associated with higher costs.

#### 5.3.7 The dynamic impact of the parameter $F_{3}$

**Fig. 18:**
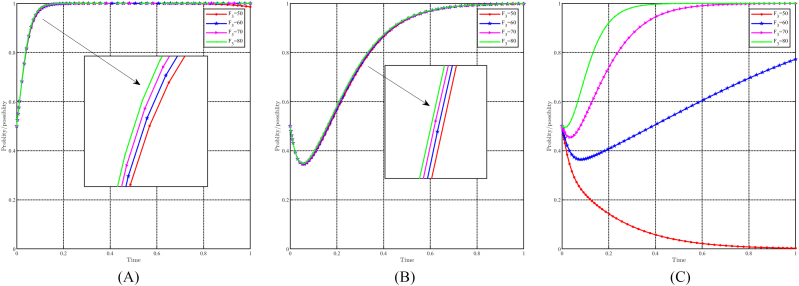
Impact of $F_{3}$ on evolutionary results and trajectories. (A) Evolution of local government. (B) Evolution of capital. (C) Evolution of people.

When $F_{3}$ takes values of 50, 60, 70, and 80, with all other parameters held constant, the effect of " $F_{3}$" on evolutionary results and paths is shown in Fig. 18A, B and 18C. In general, it can be expected that the evolutionarily stable point $E_{8}\left(1,1,1\right)$ will evolve into $E_{7}\left(1,1,0\right)$ as $F_{3}$ decreases. Changes in $F_{3}$ have minimal impact on the government and capital, while a higher $F_{3}$ value accelerates their evolutionary process. Conversely, for the people, changes in $F_{3}$ have a positive effect on their tendency toward positivity strategy. A higher f3 value increases the probability of choosing a positive strategy, accelerates the evolutionary speed, and reduces the hesitation time. Only when $F_{3}\leq 50$, the people will choose the non-positivity strategy. In particular, $F_{3}$ represents the future gains that the people can achieve by choosing positivity strategy and thus has very little effect on the strategic choices of the government and the capital. The people attach greater importance to future gains, as they can motivate changes in lifestyle and work practices if appropriately incentivized.

#### 5.3.8 The dynamic impact of the parameter $R_{21}$

**Fig. 19:**
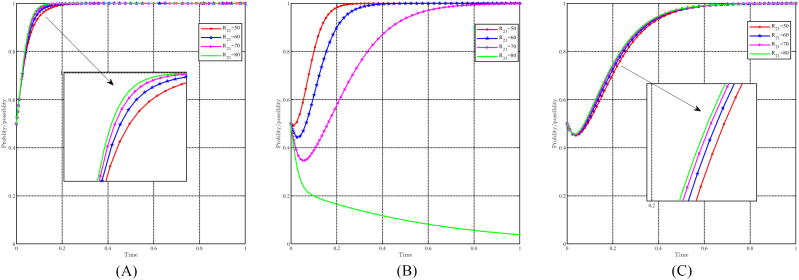
Impact of $R_{21}$ on evolutionary results and trajectories. (A) Evolution of local government. (B) Evolution of capital. (C) Evolution of people.

When $R_{21}$ takes values of 50, 60, 70, and 80, with all other parameters held constant, the effect of " $R_{21}$" on evolutionary results and paths is shown in Fig. 19A, B and 19C. In general, it can be expected that the evolutionarily stable point E8(1, 1, 1) will evolve into E_6_(1, 0, 1) as $R_{21}$ increases. Changes in $R_{21}$ have minimal impact on the government and the population, while higher values of $R_{21}$ accelerate their pace of evolution. Conversely, for the capital, changes in $R_{21}$ negatively affect their tendency to choose support strategy. A smaller $R_{21}$ increases the probability that the capital will choose a support strategy and accelerates its evolutionary speed. Once $R_{21}\geq 80$, the capital refrains from supporting the strategy. In particular, $R_{21}$ represents the benefits that the capital derives from the resource-based industry. When $R_{21}$ is too high, the capitalist finds it difficult to give up these benefits, which hinders his support for the upgrading of the industrial structure and green development.

#### 5.3.9 The dynamic impact of the parameter $R_{22}$

**Fig. 20:**
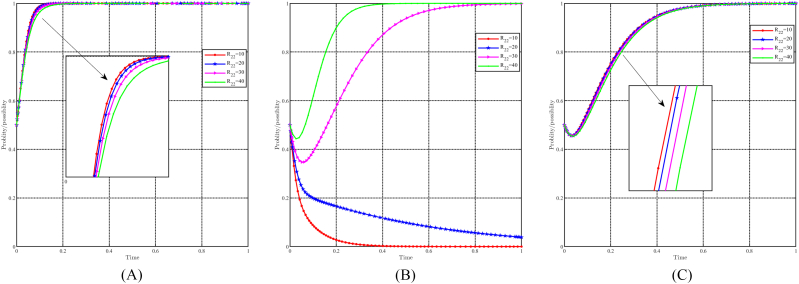
Impact of $R_{22}$ on evolutionary results and trajectories. (A) Evolution of local government. (B) Evolution of capital. (C) Evolution of people.

When $R_{22}$ takes values of 10, 20, 30, and 40, with all other parameters held constant, the effect of " $R_{22}$" on evolutionary results and paths is shown in Fig. 20A, B and 20C. In general, it can be expected that the evolutionarily stable point $E_{8}\left(1,1,1\right)$ will evolve into $E_{6}\left(1,0,1\right)$ as $R_{22}$ decreases. For the government and the people, the impact of changes in $R_{22}$ is minimal, while lower values of $R_{22}$ accelerate their evolutionary progress. Conversely, for capital, changes in $R_{22}$ have a positive effect on their choice of support strategy. When $R_{22}\geq 30$, capital chooses support strategy, thus accelerating evolution when $R_{22}$ is greater. Conversely, when $R_{22}\leq 20$, capital choose the non-support strategy, and a faster evolution speed when $R_{22}$ is smaller. In particular, $R_{22}$ represents the income earned by capital in the cultural tourism industry. Although the upgrading of industrial structure and green development should prioritize social benefits and prospects, the current income also affects the capital's decision.

#### 5.3.10 The dynamic impact of initial willingness

**Fig. 21:**
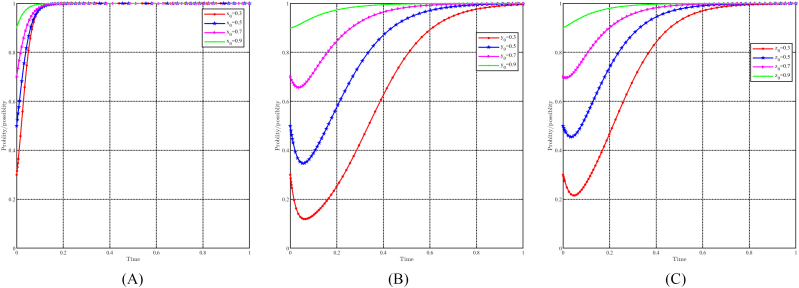
Impact of initial willingness on evolutionary results and trajectories. (A) Evolution of local government. (B) Evolution of capital. (C) Evolution of people.

Fig. 21A, 21B and 21C illustrate the effect of initial willingness on evolutionary results and trajectories by setting $x_{0}$ = 0.3,0.5,0.7,0.9, $y_{0}$ = 0.3,0.5,0.7,0.9, and $z_{0}$ = 0.3, 0.5, 0.7, 0.9 while holding other parameters constant. The figure shows that the evolutionarily stable point $E_{8}\left(1,1,1\right)$ remains constant despite variations in initial willingness. This implies that the evolutionary outcomes are not directly impacted by initial willingness. However, it does have an effect on the evolutionary trajectories, suggesting that although the final stable state remains unchanged, the approach to this state may differ depending on the initial willingness values. Stronger initial willingness promotes the choice of incentive strategies for local governments, accelerating the evolutionary process. Similarly, for capital and the people, stronger initial willingness promotes the choice of support and positivity strategy and reduces the time required to reach evolutionary stable point. Initial willingness exerts a greater influence on capital than on the people, while its influence on the government is relatively minimal.

## 6 Policy implications

The upgrading of industrial structures is a key prerequisite for achieving sustainable development. The active participation strategy of enterprises and individuals plays an important role in this transformation process. The results of this study show that several key factors influence industrial structure upgrading and green development, namely the costs associated with changing the lifestyles and production methods of both people and capital, government subsidies, future benefits for people, current benefits for capital engaged in resource-based and cultural tourism industries, and the probability of success in industrial structure upgrading. Consequently, this paper presents the following policy implications.

The government should actively strengthen the promotion of high-quality development in the cultural tourism industry, and effectively stimulate and mobilize the enthusiasm, initiative and creativity of investors to invest in this sector. To encourage investors to adopt a “support” strategy, the government should introduce stricter subsidies and penalties. Differentiated financial subsidies should be provided based on the characteristics of investors and enterprises, while punitive measures should be imposed on non-compliant enterprises to strengthen their inclination toward the “support” strategy. In addition, the government should create a conducive environment for upgrading the industrial structure and engage in cooperative efforts. This is especially important in resource-based regions that rely mainly on traditional industries. It is imperative to accelerate the transformation of government functions by emphasizing innovation within the government system, adapting to the needs of regional industrial transformation and upgrading, and shifting government management from a regulatory focus to a service-oriented approach, while maintaining the decisive role of the market in resource allocation [48].

The government should establish a comprehensive subsidy information application and disclosure system. This system should facilitate comprehensive monitoring by investors and the public, reduce information asymmetry among the three stakeholders, and effectively deter speculative practices. By improving the government's credibility and strengthening its macro-control capabilities, such measures can be implemented.

Capital should improve its investment structure by gradually shifting its investment focus from traditional labor-intensive and resource-based industries to high-tech industries as well as cultural and tourism industries [49]. This shift should be accompanied by efforts to promote energy conservation and emission reduction, and ultimately promote new competitive advantages within the industry. Attention should also be paid to the clustering effect of investment and to improving the efficiency of industrial investment.

All stakeholders should prioritize managing the relationship between future development and current risks. This involves expanding the benefits associated with future development while mitigating the risks associated with transformation in the present. It is crucial to strengthen the commitment of both capital and people to the transformation process. In addition, effective measures should be taken to provide all necessary guarantees and promote a high level of innovation throughout the industrial upgrading process.

## 7 Conclusions, limitations and research directions

### 7.1 Conclusions

To address the crucial issue of industrial upgrading, this research uses an evolutionary game model to investigate the Evolutionarily Stable Strategies (ESSs) of local governments, capital, and the people. After an analysis of the asymptotic stability demonstrated by each individual player and the overall stability of the three-player evolutionary game system, we have identified six distinct ESSs, each associated with specific stability conditions. We then perform numerical simulations to elucidate the impact of these six ESSs and the key parameters on the tripartite ESS. The main results of this study can be briefly summarized as follows.

With regard to the six ESSs and their stable conditions, this study identifies six ESSs and their corresponding stable conditions. When local governments choose incentive strategies, both capital and the people tend to support upgrading the industrial structure and promoting green development in the regional economy respectively. However, their specific strategy choices depend on their respective benefits and payoffs.

The choice of capital strategy depends on a comparative analysis of transition costs versus immediate benefits. More precisely, given that the immediate benefits of the capital's transition to the culture and tourism industry are smaller, coupled with the incurrence of additional transition costs, the importance of future benefits and government subsidies becomes pronounced. On the one hand, it is incumbent upon the government to provide the capital with multifaceted support, especially in the form of economic aid, to ensure that the financial burden is eased throughout the process of improving the industrial structure [50]. The capital can make further use of political support to secure financial assistance in the initial stages of industrial structure improvement [51]. Conversely, both the government and capital should strengthen investment and development in the cultural and tourism sectors, and cultivate a favorable socio-economic environment conducive to the ecologically conscious development of the regional economy [52]. By improving the quality of scenic attractions, cultural events, and tourism services, a diverse array of attractions can be established, thereby exerting a magnetic attraction for increased tourist traffic and investment [53]. In addition, such initiatives will increase the future income of capital engaged in the development of the cultural and tourism industries, thus promoting a favorable development outlook for the structural transformation of the sector.

People's choice of strategies depends primarily on government subsidies and anticipated benefits. The transition to a greener regional economy promises not only a better living environment but also a healthier working environment. Therefore, when substantial government subsidies and future benefits are available, people tend to adopt positive strategies. Therefore, the government must step up its incentive efforts, including increased financial subsidies and the establishment of more enticing career development initiatives for those working in the cultural tourism sector. In addition, concerted efforts by both the government and capital investors are needed to disseminate information and facilitate promotional campaigns that highlight the lasting benefits of upgrading the industrial structure, along with the associated environmental benefits [54]. This concerted approach is designed to generate public enthusiasm for proactive involvement in the development of the cultural and tourism industries. It is incumbent upon government agencies and investors alike to promote the robust development of the cultural and tourism sector and to continually refine its prospective economic benefits [55].

The study identified the impact of pivotal parameters on both evolutionary results and trajectories. The costs associated with the adoption of altered lifestyles and production methods by people and capital, government subsidies, prospects for individuals, and immediate gains for capitalists in both resource-dependent and cultural tourism industries stand out as pivotal factors influencing evolutionary outcomes and trajectories. However, the probability that the government will implement a carbon tax and its initial willingness to do so only affect evolutionary trajectories. The probability of carbon tax implementation and the initial predisposition of the three stakeholders do not influence the evolutionary result but rather influence the evolutionary trajectory to varying degrees. In a holistic sense, the government should seek to minimize the costs associated with adopting positive strategies by increasing the direct economic subsidies to both the capital and the people. At the same time, serious efforts should be made to promote the upgrading of the industrial structure. This is especially important for realizing high-quality economic development and weaning the regional economy from its dependence on resource-oriented sectors, especially the coal industry, thus promoting green regional development along with improved production and lifestyle paradigms [56,57]. These aspirations for a healthier lifestyle and environment unite all stakeholders [58]. The convergence of economic and environmental gains has a significant impact on future returns. In particular, the probability of success in upgrading the industrial structure is the key factor shaping the strategic choices of all entities. Consequently, the formation of a comprehensive collaborative framework among local governments, capital, and the people becomes imperative. Such cooperation, backed by enhanced resources and support, will increase the confidence and motivation of capital and the people, thus increasing their commitment to the endeavor. Ultimately, this synergy will culminate in more lucrative future gains, thereby improving both the success rate and the benefits of industrial structure upgrading.

### 7.2 Limitations

This study examines the strategic behavior within a regional industrial upgrading framework involving local governments, capital, and people. It also examines the impact of relevant variables on the equilibrium of the system. However, this study has certain limitations.

First, it operates under a relatively idealized premise, assuming the autonomy of local governments, capitalists, and the general public as stakeholders. However, real-world dynamics encompass a spectrum of stakeholders, including the central government and civil society organizations, making it difficult for this paper to capture the entirety of this diverse spectrum.

Second, this study only models and analyzes the overarching scenario within Shanxi Province, and overlooks the potential influence of cultural differences and geographical characteristics inherent to individual cities on the process of upgrading industrial structure. Different regions may differ in their adoption of upgrading strategies due to factors including historical context, available resources, and their existing industrial base.

Finally, industrial structural upgrading encounters influence not only internal system dynamics but also external conditions encompassing the global landscape and economic cycles. Unfortunately, this paper does not consider the implications of changes in the international environment.

### 7.3 Research directions

An upcoming research objective is to explore methodologies for constructing a more comprehensive model that incorporates the interests of multiple stakeholders within the complex framework of the real world. At the same time, attention will be paid to the integration of technical factors, including industrial innovation and the digital transformation of the culture and tourism sector.

In addition, China is currently undergoing a period of industrial upgrading, which brings new challenges all the time. This inherent complexity hinders the construction of real-time model simulations. Therefore, the future research path requires sustained attention to formulating real-time systems that exploit the synergy of data mining, machine learning, and related technologies.

The socio-cultural and environmental dynamics of different cities, including cultural values and the cultivation of human capital, exert a substantial influence. Future research could delineate the impact of these aspects on decisions and strategies for industrial upgrading.

The greening of regional economies and concomitant industrial upgrading are often shaped by cross-regional and international factors. One avenue for future investigation lies in exploring the interconnectedness of elements such as global supply chains and international trade policies and their impact on intra-regional decision-making and development outcomes.

## Data availability statement

Data included in article/supp. material/referenced in article.

## CRediT authorship contribution statement

**Fuqiang Hu:** Data curation, Formal analysis, Writing – original draft, Writing – review & editing. **Bodong Song:** Conceptualization, Methodology, Validation, Writing – review & editing. **Chengmeng Li:** Conceptualization, Data curation, Formal analysis, Methodology, Writing – review & editing. **Kwisik Min:** Supervision.

## Declaration of competing interest

The authors declare that they have no known competing financial interests or personal relationships that could have appeared to influence the work reported in this paper.
